# Low Sensitivity of the Formol-Ethyl Acetate Sedimentation Concentration Technique in Low-Intensity *Schistosoma japonicum* Infections

**DOI:** 10.1371/journal.pntd.0000386

**Published:** 2009-02-24

**Authors:** Tore Lier, Gunnar S. Simonsen, Tianping Wang, Dabing Lu, Hanne H. Haukland, Birgitte J. Vennervald, Maria V. Johansen

**Affiliations:** 1 Department of Microbiology and Infection Control, University Hospital of North Norway, Tromsø, Norway; 2 Department of Microbiology and Virology, Faculty of Medicine, University of Tromsø, Tromsø, Norway; 3 Norwegian Institute of Public Health, Oslo, Norway; 4 Anhui Institute of Parasitic Diseases, Wuhu, People's Republic of China; 5 Department of Infectious Diseases, Faculty of Medicine, Imperial College, London, United Kingdom; 6 DBL- Centre for Health Research and Development, Faculty of Life Sciences, University of Copenhagen, Copenhagen, Denmark; Swiss Tropical Institute, Switzerland

## Abstract

**Background:**

The endemic countries are in a diagnostic dilemma concerning *Schistosoma japonicum* with increasing difficulties in diagnosing the infected individuals. The formol-ethyl acetate sedimentation concentration technique is preferred by many clinical microbiology laboratories for the detection of parasites in stool samples. It is potentially more sensitive than the diagnostic methods traditionally used.

**Methodology/Principal Findings:**

We evaluated the technique for detection of low-intensity *S. japonicum* infections in 106 stool samples from China and used a commercial kit, Parasep Midi Faecal Parasite Concentrator. One stool sample and one serum sample were collected from each person. As reference standard we used persons positive by indirect hemagglutination in serum and positive by Kato-Katz thick smear microscopy (three slides from a single stool), and/or the hatching test. We found the sedimentation technique to have a sensitivity of only 28.6% and specificity of 97.4%.

**Conclusion/Significance:**

This study indicates that the sedimentation technique has little to offer in the diagnosis of low-intensity *S. japonicum* infections, at least when only a single stool sample is examined.

## Author Summary

*Schistosoma japonicum* is parasitic fluke (worm) found in China, Indonesia and the Philippines. A lot of effort has been put into combating the parasite, and the result has been a large drop in the number of infected people over the last decades. The average infected person also now has few worms, and hence excretes few eggs in stool. This has made it increasingly difficult to get a correct diagnosis by the diagnostic tests traditionally used. Tests based on detecting eggs in stool can be false-negative and tests detecting antibodies can be false-positive due to persisting antibodies or antibodies from other worm infections. Hence there is a need for new diagnostic strategies. Formol-ethyl acetate sedimentation concentration is a technique for detecting eggs in stool by microscopy, but has not to our knowledge been evaluated for *S. japonicum*. We compared the technique, using a single stool sample and a commercial preparation kit, with three tests traditionally used in the endemic countries (Kato-Katz thick smear, hatching test and indirect hemagglutination antibody detection). The sedimentation technique detected disappointing few positives and seems not to be an advantage in the diagnosis of low-intensity *S. japonicum* infection, compared to the traditionally used tests.

## Introduction

Schistosomiasis japonica is still a major public health problem, especially in China, despite great achievements during the past 50 years in controlling this parasitic disease. Diagnosis is a key for decision-making, both on individual and community levels. The current epidemiologic situation in the *Schistosoma japonicum*-endemic countries (China, the Philippines and Indonesia) has made the use of many common diagnostic assays problematic [1]. Low-intensity infections reduce the sensitivity of tests which demonstrate eggs in stool such as the Kato-Katz thick smear [2] and the hatching test [3], especially when only a single stool sample is available for examination. Antibody detection tests suffer from low specificity, often due to persistent antibodies from previously treated infections [4]–[6].

Stool concentration techniques, especially formol (formalin)-ether or formol-ethyl acetate sedimentation concentration is the preferred method for detection of helminth eggs and protozoa cysts in many clinical parasitology laboratories [7]–[9], although to a lesser extent in Africa and Asia. The formol-ethyl acetate sedimentation concentration technique (subsequently termed ‘sedimentation technique’) is one of the methods recommended in the Clinical and Laboratory Standards Institute (formerly NCCLS) guidelines [10] and the Cumitech guidelines [11] for recovery of parasites from the intestinal tract. The sedimentation technique processes a relatively large stool sample and is thus theoretically more sensitive than alternative techniques in low-intensity helminth infections. To our knowledge there are no published data evaluating this method for the detection of *S. japonicum*. As the sedimentation technique is more resource-demanding than the Kato-Katz technique in terms of equipment and workload, the method should substantially improve the diagnostic yield in order to be cost-effective. The primary aim of this study was to assess whether the sedimentation technique could be a sensitive alternative to the tests traditionally used in the endemic areas, and hence potentially play a role in a diagnostic algorithm of the control programs. Additionally, as diagnosing light infections is a challenge in the individual patient too, information from this study might find interest in travel medicine (where the sedimentation technique is traditionally used) and at clinics in endemic areas.

## Materials and Methods

### Study area, population and ethical considerations

Stool samples and sera were collected in the villages Guanghui and Heping in Laozhou township, located on an island in the Yangtze River in Tongling county, Anhui province, China. Persons qualified for the local control program were recruited. Ethical approval from the village leadership, the Scientific Committee at Anhui Institute of Parasitic Diseases and Tongling County Health Bureau was obtained. All study subjects were informed about the study but due to widespread scepticism to signing a consent form, oral informed consent was used. Oral consent was witnessed by representatives from the village leadership and the name of the participant was then registered by the research team. Oral consent was approved by the village leadership and the Scientific Committee. The study was embedded in the long ongoing schistosomiasis control program, which is based on serum and stool samples, usually performed as an initial sero-diagnosis and followed by stool diagnosis of sero-positive individuals [1],[12],[13]. Renouncing the study did not exclude anyone from the regular control program. Participants found positive were treated with a single oral dose of praziquantel (40 mg/kg).

### Field and laboratory procedures

One stool sample and one serum sample were collected from all participants. The sedimentation technique, Kato-Katz thick smear and hatching test were performed on the same stool sample. Parasep Midi Faecal Parasite Concentrators are closed, single use tubes with built-in filter. They were pre-filled with 6 ml 10% buffered formalin, one drop Triton-X and 2 ml ethyl acetate and used for the sedimentation technique in accordance with the manufacturer's instruction sheet (DiaSys Europe Ltd., Berkshire, United Kingdom). A teaspoon was used to measure an amount of stool equivalent to 1 g. A single slide was examined per sample, using a large coverslip (24×50 mm), going through the whole slide at 100× magnification. Previous test results were not known to the microscopist.

The hatching test was done by adding approximately 30 g stool into a container with a coarse metal mesh, flushing the fine material into a fine-meshed nylon bag and then washing until the water was clear. The content of the bag was transferred to a triangular flask containing 300 ml non-chlorinated water. The flasks were examined in strong light by two members of the staff for the presence of swimming miracidia after 4, 6, 8 and 24 hours [5].

Kato-Katz thick smear was performed as previously described [5],[14], using nylon screens and plastic templates. Three slides (41.7 mg each) were prepared from a single stool from each person and examined within two weeks. Positive slides were confirmed by a second microscopist.

Sera were collected in capillary tubes. Antibody detection was done by IHA, using a kit commercially manufactured at Anhui Provincial Institute of Parasitic Diseases as described by Zhou *et al.* [6]. The test was considered positive if a positive reaction appeared at a titre ≥1∶10.

## Results

A total of 144 samples were selected from a larger collection (1360 samples) based on the result of Kato-Katz thick smear and hatching test (approximately one-third positive). However, 38 samples were discarded because of inadequate sample size or missing results thus leaving 106 samples for further analysis. This study population of 106 persons consisted of 56% males and 44% females aged 7–76 years (mean 39 years). The number of positive persons for each test was 47 (44%) for IHA, 19 (18%) for Kato-Katz thick smear, 27 (26%) for hatching test and 10 (9%) for the sedimentation test. A person was considered positive by the “reference standard” if IHA was positive together with Kato-Katz positive and/or hatching test positive. A total of 28 persons (26%) were reference standard positive, including 15 who were hatching positive/Kato-Katz negative and 5 who were Kato-Katz positive/hatching negative. Eight persons were positive in both tests. Results from Kato-Katz thick smear, the only fully quantitative test in this study, showed that the majority of the samples had low egg count, and hence probably were low-intensity infections. In the reference standard positive group there were 7 samples containing <40 egg per gram of stool (EPG), 5 containing 40–99 EPG and 1 sample containing 368 EPG.

When we used this reference standard, we found a sensitivity of 28.6% and a specificity of 97.4% for the sedimentation technique (Table 1). The 2 individuals who were positive by the sedimentation technique, but reference standard negative (see Table 1), were single positives in IHA and Kato-Katz, respectively. The number of *S. japonicum* eggs on the sedimentation technique slides ranged from 1 to 18 (median 2.5).

**Table 1:** Results of *S. japonicum* microscopy of stool samples by the formol-ethyl acetate sedimentation concentration technique.

Sedimentation technique	Reference standard		Total
	Positive	Negative	
Positive	8	2	10
Negative	20	76	96
Total	28	78	106

‘Reference standard’ refers to individuals positive by indirect hemagglutination (IHA) and positive by Kato-Katz microscopy and/or the hatching test.

Out of the 76 persons who were both reference standard and sedimentation technique negative, there were 18 only positive with IHA, 5 only with Kato-Katz and 4 with a positive hatching test only.

There were 8 samples positive for other helminth eggs with Kato-Katz (*Trichuris trichiura* = 7, *Ascaris lumbricoides* = 1) and 7 samples positive with the sedimentation technique (*T. trichiura* = 3, *A. lumbricoides* = 1, hookworm = 2, *Hymenolepis diminuta* = 1).

## Discussion

In this study we found that the sedimentation technique, when a commercial kit (Parasep Midi Faecal Parasite Concentrator) and a single stool sample was used, had a disappointingly low sensitivity in diagnosing low-intensity *S. japonicum* infections. However, the number of samples tested is low and further studies are needed to confirm the results.

The hatching test, Kato-Katz thick smear and serum antibody detection methods such as IHA are all commonly used in China, but to our knowledge there are no published data evaluating the sedimentation technique for detection of *S. japonicum*. Several authors have compared sedimentation techniques and Kato-Katz in the diagnosis of *S. mansoni* with divergent conclusions as to which has the highest sensitivity. The two largest comparative studies conclude that the sensitivity of 2 or 3 Kato-Katz slides from a single stool is superior to the sedimentation technique [15],[16]. However, in detecting light infections, several authors found the sedimentation technique to be more successful [8],[16].

In our opinion the performance of the sedimentation technique for *S. mansoni* or other helminths cannot automatically be extrapolated to *S. japonicum* mainly because *S. japonicum* eggs are round and lack conspicuous characteristics. They are often surrounded by stool material. Hence a particularly watchful eye is needed to detect low-intensity infections.

The sedimentation technique had a low sensitivity in this study. Even if we used a positive Kato-Katz or a positive hatching test as the only positive reference standard criterion, regardless of IHA, the test properties of the sedimentation technique remained almost the same with a sensitivity of 23.7% and specificity 98.8%. There may be several explanations for the low sensitivity. When the egg output is very low, it will be a matter of chance whether there is an egg on the slide or not. This may also explain why only few in the reference standard group were positive for both Kato-Katz and hatching. Knight *et al.* [8] and Ebrahim *et al.* [16] both calculated that half of all *S. mansoni* eggs were lost in the sedimentation technique procedure compared to Kato-Katz. Examination of more than one sedimentation technique slide from each sample would most likely increase the sensitivity, but also be very time-consuming compared to Kato-Katz. We used approximately 15 min to examine each sedimentation technique slide, while an experienced technician uses approximately 3 min on each Kato-Katz slide. It has been shown that the number of *S. japonicum* eggs may vary between consecutive stool samples from the same person, favouring examination of repeated samples [17]. Collecting repeated stool samples in large control programs is often not feasible [18],[19]. However, in handling individual patients, such as in travel medicine and in clinics in endemic areas, examining repeated stool samples is easier to accomplish, and are indeed often recommended. In the present study we examined a single stool sample, and hence could not determine the value, especially in terms of sensitivity, of repeated sampling. The sedimentation technique gives the opportunity to examine for intestinal protozoa as well. Hookworm eggs clear rapidly in the Kato-Katz procedure and will usually not be visible unless the smears are examined soon after preparation [14],[20],[21]. We found 2 hookworm-positive samples by the sedimentation technique and none by Kato-Katz. The ability to detect a broad range of intestinal parasites is usually important when taking care of individual patients, while both intestinal protozoa and hookworm are in most cases of secondary interest in *S. japonicum* control programs. The sedimentation technique had high specificity in this study, and even the two “false positive” sedimentation technique samples might possibly be the result of a false negative reference standard when interpreted in light of the aforementioned low sensitivity of Kato-Katz and hatching tests, especially when only a single stool sample is examined [5],[6],[17],[18].

We found the Parasep Midi Faecal Parasite Concentrator kit to be very practical for field sampling and preparation with limited hands-on time required. The material costs may be higher than with the conventional open sedimentation technique. Unfortunately there are, to our knowledge, no peer-reviewed papers published that directly compare the Concentrator kit with a conventional open sedimentation technique. An unpublished evaluation has been performed by Kettelhut *et al.* at the Hospital for Tropical Diseases, London (personal communication, available through the manufacturer and provided here as Text S1), where the Concentrator kit shows similar results as a standard open sedimentation technique. However, generalizing the results using this kit to other variations of the sedimentation techniques should be avoided.

Based on the findings in this study we conclude that the formol-ethyl acetate sedimentation concentration technique, using Parasep Midi Faecal Parasite Concentrators, offers no advantages compared to Kato-Katz and hatching in low-intensity *S. japonicum* infections. The sedimentation technique had low sensitivity and is relatively time-consuming. Clinical laboratories using the sedimentation technique might also consider using additional tests to diagnose imported *S. japonicum* infections. However, if examining repeated samples is feasible, it will likely increase the sensitivity.

## Supporting Information

Alternative Language Abstract S1 Translation of the Abstract into Chinese by Dabing Lu.
(0.06 MB PDF)

Text S1 Evaluation of Parasep Faecal Parasite Concentrator.
(3.50 MB PDF)
