# Supplementary material for: Low Sensitivity of the Formol-Ethyl Acetate Sedimentation Concentration Technique in Low-Intensity Schistosoma japonicum Infections
Source: PLoS Negl Trop Dis. 2009 Feb 24;3(2):e386. doi: 10.1371/journal.pntd.0000386 (PMC2638014; doi:10.1371/journal.pntd.0000386)
Supplement: Alternative Language Abstract S1 — Translation of the Abstract into Chinese by Dabing Lu. (0.06 MB PDF) [file pntd.0000386.s001.pdf]

甲醛-乙酸沉积法在诊断血吸虫低感染度人群的低灵敏性

摘要

背景：在血吸虫病流行国家，随着血吸虫感染者检出难度日益增加，有关血吸虫病诊断已处困境。基于甲醛-乙酸沉积法可能较传统诊断方法敏感，很多临床微生物实验室乐于采用前法进行粪样虫卵检查。

方法及重要发现：我们采用一种商业诊断试剂盒（Parasep Midi Faecal Parasite Concentrator）对取自中国低度感染者的 106 份粪样进行了检查，并同时对每个感染者采集了血清标本。以间接血凝阳性和 Kato-Katz 涂片镜检法（一份粪样 3 张涂片）、和/或粪孵法阳性作为参照标准，我们发现此种沉积法灵敏度和特异度分别是 28.6%和 97.4%。

结论及意义：研究提示此种沉积诊断法在仅采集一次粪样时，对低度血吸虫感染者检出意义不大。
