# Supplementary material for: Low Sensitivity of the Formol-Ethyl Acetate Sedimentation Concentration Technique in Low-Intensity Schistosoma japonicum Infections
Source: PLoS Negl Trop Dis. 2009 Feb 24;3(2):e386. doi: 10.1371/journal.pntd.0000386 (PMC2638014; doi:10.1371/journal.pntd.0000386)
Supplement: Text S1 — Evaluation of Parasep Faecal Parasite Concentrator. (3.50 MB PDF) [file pntd.0000386.s002.pdf]

Evaluation of Parasep® Faecal Parasite Concentrator

M. Kettelhut, A. Moody, H. Edwards and P.L. Chiodini

Hospital for Tropical Diseases Tooley Street, London. England.

Summary

The use of a concentration method by clinical laboratories is essential to increase the sensitivity of finding ova, cysts and larvae in faecal specimens as they may be to scanty to be seen by direct microscopy. The Ridley-Allen modified formol ether sedimentation technique is the method of choice for routine use by most clinical laboratories. This procedure utilises filtration of a faecal suspension followed by solvent extraction and centrifugation. It requires several pieces of apparatus and can present potential COSHH problems.
Parasep, a commercial kit for faecal concentration, developed by the company Intersep, is an enclosed, single use disposable system which minimises the pieces of apparatus used and is a less hazardous procedure of comparable efficiency to the standard method.
A comparison between the open and enclosed systems using ether or ethyl acetate with Triton-X as the solvent extraction gave a similar recovery of parasites and negligible interference in the amount of debris present in the deposit. There was, however, a significant increase in the recovery of certain parasites namely *Taenia species* and *Hymenolepis nana*, and in some specimens stored in 10% formalin at 4°C containing ova of *Ascaris lumbricoides* and *Toxocara canis*, when ethyl acetate was used.

Introduction

The microscopic examination of faeces is essential for the recognition and identification of intestinal parasites. Direct microscopy, although useful for the observation of motile protozoan trophozoites and the examination of cellular exudate, is not recommended solely for the routine examination of faeces with suspected parasitic infections. In order to maximise the numbers of organisms detected, a concentration method is essential to increase the possibility of recovering ova, cysts and larvae which may be too scanty to detect by direct microscopy alone.

The Ridley-Allen (RA) modified formol ether technique is the procedure recommended for clinical laboratories for the routine diagnosis of parasitic infections and is the technique used routinely by the Department of Clinical Parasitology, Hospital for Tropical Diseases in London. This method utilises ether or ethyl acetate as an extractor of fat and debris from faeces after filtration and leaves the parasites in a sediment at the bottom of the tube after centrifugation. The advantages of this method are that it will recover most ova, cysts and larvae and retains their morphology thus facilitating identification.

The method can also be used on samples which have been preserved in formalin, sodium acetic acid formalin (SFA) and polyvinyl alcohol (PVA), but has the disadvantage of destroying trophozoite stages and distorting cellular exudate, liquid faeces do not concentrate well thus it is necessary in these cases to examine the stool by direct microscopy.

This study compares the conventional open method for modified Ridley-Allen concentration technique used at the Hospital for Tropical Diseases; with Parasep, a commercial kit developed by Intersep[1] (DiaSys Europe), Brook House, Molly Millars Bridge, Wokingham, Berkshire, RG41 2WY, England, which is an enclosed, single use, disposable system for the modified RA sedimentation technique.

Materials and Methods

The Parasep Faecal parasite concentrator employs the principal of the Ridley-Allen formol-ether sedimentation technique in an enclosed system.

It consists of a mixing chamber in which the faeces is mixed with the 10% formalin. The ether or ethyl acetate is added (1 drop of Triton-X is added to the mixture when ethyl acetate is used as it helps to break up the faecal matter) and Parasep is immediately sealed by screwing the filter/thimble sedimentation cone onto the mixing chamber. The seal is an air/liquid seal which prevents the release of biohazardous material. There is also a safety lock to ensure that the mixing chamber and filter thimble are removed together for safe disposal.

The mixture is vortexed and Parasep is then inverted to allow the mixture to be filtered through the filter thimble. The filter thimble is made from high density polyethylene and has a 2 stage filtration matrix which means that the large particles are rejected without occluding the 425 µm pores. There is also a debris trap so that rejected particles are trapped to prevent extrusion into the sedimentation cone.(*see Fig.1*). Parasep is then centrifuged at 3000 rpm for 1 minute. The mixing chamber and filter thimble are unscrewed and discarded.

[1] Now DiaSys Europe, 5 the Sapphire Centre, Fishponds Road, Wokingham, Berkshire England. RG41 2QL

Like the conventional Ridley-Allen sedimentation method, there is a ether/ethyl acetate layer, fatty plug, formalin and sediment, The fatty plug is loosened and the supernatant discarded. The deposit is examined for ova, cysts and larvae.

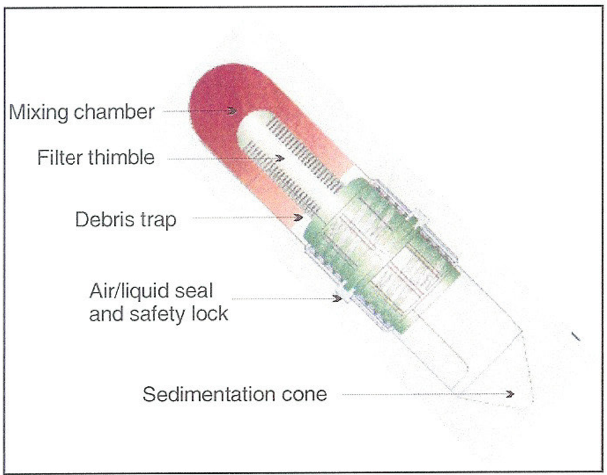

Fig. 1

The standard Ridley-Allen formol-ether concentration use as by HTD follows the method as described as in Reference 1.

100 faecal samples containing previously diagnosed ova, cysts or larvae were examined by the Ridley Allen sedimentation technique as used by the Hospital for Tropical Diseases and by the Parasep. Ether and ethyl acetate with 1 drop of Triton X, were compared in parallel as the lipid extracting agent on both techniques. The faecal samples were divided into the following categories:

1. 26 faecal samples containing ova, 21 of which contained only 1 species of helminth and 5 contained
2 or 3 species of helminths.
2. 24 faecal samples containing protozoan cysts or oocysts; 15 of which contained only one species of protozoa and 9 contained 2 or more protozoa.
3. 50 containing no ova, cysts or larvae.

Some faecal samples were preserved in formalin and stored in 1ml aliquots at 4°C. The remainder of the specimens were fresh and unpreserved and examined directly.

Results

100 stool specimens were examined in parallel by both Ridley-Allen open method and enclosed Parasep techniques using ether and ethyl acetate.

The number of ova and larvae were counted per total deposit and the number of cysts were counted per field using a x40 objective. Where there was more than 1 specimen containing the same parasite, the average number of parasites present was calculated.

Group 1: Specimens containing ova and larvae

1a. 21 specimens containing ova and larvae of 1 species of helminth (*Fig 2)*

	Average number of ova per deposit			
	Open Ridley-Allen concentration method		Enclosed Parasep faecal concentrator	
Helminth species	Ether	Ethyl acetate	Ether	Ethyl acetate
Ascaris lumbricoides x2	114	108	116	148
Hookworm species x4	95	123	121	132
Trichuris trichiura x3	11	6	22	15
Rhabditiform larvae of Strongyloides stercoralis x1	20	20	18	16
Filariform larvae of Strongyloides stercoralis x3	28	18	40	27
Toxocara canis x1	8	183	12	198
Trichostrongylus species x1	4	2	4	2
Taenia species x2	26	62	17	60
Hymenolepis nana x4	13	96	14	75

Fig. 2

1b. 5 mixed helminth infections (*Fig 3*)

	Average number of ova per deposit			
	Open Ridley-Allen concentration method		Enclosed Parasep faecal concentrator	
Helminth species	Ether	Ethyl acetate	Ether	Ethyl acetate
Ascaris lumbricoides and	185	130	145	115
Trichiuris trichiura	11	3	9	1
Hookworm species and	6	10	6	7
Schistosoma mansoni	9	10	12	10
Ascaris lumbricoides,	100	300	186	273
Trichuris trichiura and	17	23	16	36
Hookworm species	12	70	50	60
Ascaris lumbricoides,	34	160	38	148
Trichuris trichiura and	16	6	10	16
Hymenolepis nana	3	4	2	2
Ascaris lumbricoides,	0	4	0	2
Hookworm species and	4	6	5	4
Schistosoma mansoni	8	8	10	17

Fig. 3

A comparable recovery of parasites was noted in both methods. However, a significant increase was noted in the recovery of ova of *Taenia species*, *Hymenolepis nana, Ascaris lumbricoides* and *Toxocara canis* when ethyl acetate with Triton-X as a lipid extraction agent was used compared to ether.
The ova of *Tania* species and *Hymenolepis nana* are light and on examination, it was noted that they had become trapped in the fatty flug and were discarded. The ova of *Toxocara canis* and *Ascaris lumbricoides,* in particular those in table 2, had been stored in formalin for long periods and may have altered their density.

Group 2: Specimens containing cysts (*Fig 4*)

	Average number of cysts/oocysts per field (x40 objective)			
	Open Ridley-Allen Method		Enclosed Parasep faecal concentrator	
Protozoa species	Ether	Ethyl Acetate	Ether	Ethyl Acetate
Endolimax nana x2	++	+	++	+
Entamoeba histolytica x1	+/--	+/--	+/--	+/--
Entamoeba coli x3	+/-	+/-	+/-	+/-
Chilomastix mesnili x1	+++	+++	+++	+++
Giardia lamblia x3	+/-	+/-	+/-	+/-
Cyclospora cayetanensis x3	+/-	+/-	+/-	+/-
Isospora belli x4	+	+	+	+

Fig. 4

2b. Mixed protozoa infections (*Fig 5*)

	Number of cysts/oocysts per field (x40 obj)			
	Open Ridley-Allen Method		Enclosed Parasep faecal concentrator	
Protozoa species	Ether	Ethyl Acetate	Ether	Ethyl Acetate
Entamoeba coli	+/-	+/-	+/-	+/-
Endolimax nana	+/-	+/-	+/-	+/-
Entamoeba coli	+/-	+/-	+/-	+/-
Entamoeba histolytica	+/-	+/--	+/-	+/-
Entamoeba histolytica	+/-	+/--	+/-	+/--
Iodamoeba butschlii	+/-	+/--	+/-	+/--
Cyclospora cayetanensis	+	+	+	+
Endolimax nana	+	+/-	+/-	+
Entamoeba histolytica	++	++	++	++
Endolimax nana	+	+/-	+	+/-
Entamoeba hartmanni	+	+/-	+	+/-
Chilomastix mesnili	+++	++	++	+
Endolimax nana	+	+	+	+
Entaimoeba histolytica	+	+	+	+
Entamoeba coli	+/-	+/-	+/-	+/-
Entamoeba histolytica	+	+	+	+
Iodamoeba butschlii	+	+	+	+
Entamoeba Hartmanni	+/-	+/-	+/-	+/-
Entamoeba coli	+/-	+/--	+/--	+/--

Fig. 5

A comparable recovery of parasites was noted using both methods. However, there was considerably more deposit using ethyl acetate making the cysts more difficult to see without dilution.

Cyst enumeration code

As it is impractical to count the number of cysts/oocysts per deposit, the following code was used:

+++	>10 cysts per field (x 40 objective)
++	5 - 10 cysts per field (x 40 objective)
+	1 - 5 cysts per field (x 40 objective)
+/-	1 cyst per 2 - 10 fields (x 40 objective)
+/--	<1 cyst per 10 fields

Group 3: 50 negative faeces

50 faeces were shown to to contain no ova. cvsts or larvae by the modified Ridley Allen open technique and Parasep, using both ether and ethyl acetate.

Discussion

This study compared the modified Ridley Allan concentration as an open technique used routinely by the Department of Clinical Parasitology, Hospital for Tropical Diseases with Parasep, which is an enclosed, single use, disposable system.

In order to compare the two procedures one hundred faecal samples were concentrated in duplicate by both techniques using ether or ethyl acetate as the lipid extracting agents. The faecal samples consisted of samples containing a wide range of ova, larvae, cysts and oocysts. When comparing the techniques, the most important observations considered included the recovery of parasites, the density of the deposit, ease of handling, health and safety aspects and cost.

An important factor when deciding on the parasite concentration technique to employ on faecal samples is the recovery of parasites. In this comparison, the number of ova and larvae were counted per deposit and the average number of cysts and oocyts were counted per field (due to the impractability of counting the cysts per deposit). Where more than one sample containing the same parasite was examined, the average number was calculated. Both fresh and preserved samples were examined.

Overall, the recovery of parasites by both techniques was comparable. All parasites present were detected by the two procedures and in equivalent numbers (Tables 1-4). However,a significant increase in parasite numbers was observed with certain helminth species, namely ova of *Taenia species* and *Hymenolepis nana*, when ethyl acetate along with Triton-X was uses as the lipid extraction agent.

On analysis, it was noted that when ether was used, these ova of these helminths became trapped in the fatty plug which is discarded along with the supernatant. Possible explanations could be that the ova of *Taenia species* are small and those of *H. nana* are light thus become trapped in the debris when ether is used.

The addition of Triton-X when ethyl acetate is applied as a lipid extracting agent, results in more debris in the deposit. A significant increase in parasite numbers was also noted in preserved specimens containing ova of *Ascaris lumbricoides* and *Toxocara canis* when ethyl acetate was used. A notable example was the specimen in table 2 containing ova of *Schistosoma mansoni* and *Hookworm species*, in which scanty ova of*Ascaris lumbricoides* was detected only by the ethyl acetate procedure.

A possible explanation for that particular observation is that the density of certain ova is altered when they are preserved in formalin and stored for long periods, resulting in them becoming more buoyant and becoming trapped in the fatty plug and subsequently discarded.

Although a sedimentation concentration technique maximises the recovery of most ova, cysts and lanrvae, the deposit may contain some debris which, when present in excess, could mask the presence of small parasites, especially cysts. A notable difference was observed, however, between the final deposits when using ether or ethyl acetate, the latter resulting in much thicker deposit resulting in potential obscuring of cysts.

The rapidity and safety by which the samples can be processed are factors to take into consideration when deciding on an appropriate concentration technique. The standard open Ridley-Allen sedimentation tech-ique uses several pieces of apparatus (ie. centrifuge tubes, boiling tubes, brass seive and a collection receptacle) which can be rather labour intensive to use and clean.

The Parasep technique has the advantage of being a disposable enclosed system thus minimising the number of pieces of apparatus used. This speeds up the whole procedure, makes it easier to handle and more user friendly. The use of 10% formalin as a fixative, reduces the risk of infection from bacteria and viruses. However, exposure to formalin is an irritant and ether is flammable (ethyl acetate being less flammable option), thus it is necessary to carry out this procedure in a spark proof extraction cabinet.

Parasep, however, is a totally enclosed process and has an air/liquid seal and safety lock, the seal preventing the release of hazardous material and the lock ensures that the mixing chamber and filter thimble are removed together for safe disposal after centrifugation. It is also a single use device which is discarded after use so minimising the risk of sample contamination.

Cost is an important consideration in deciding which concentration technique to employ. In the standard Ridley-Allen sedimentation technique, the initial purchase of brass filter, polycarbonate centrifuge tubes and boiling tubes is expensive but can be cleaned and recycled. Parasep is a single use, disposable device which can appear more expensive but can be equated with the time spent and cost of cleaning the pieces of equipment. On balance, there does not appear to be a cost advantage by either method.

In summary, the recovery of parasites by the open method for the Ridley-Allen sedimentation technique and Parasep, a commercial faecal parasite concentrator, is comparable. The former procedure is lower cost but it is labour intensive and has inherent health and safety hazards. Parasep, however, is an enclosed system and a single use, disposable device thus making it less hazardous and more user friendly. We consider that the Parasep used with ethyl acetate offers a safer, more user friendly approach to faecal concentration using the Ridley-Allen formol ether sedimentation method.
